# Real-life GH dosing patterns in children with GHD, TS or born SGA: a report from the NordiNet® International Outcome Study

**DOI:** 10.1530/EJE-16-1055

**Published:** 2017-05-18

**Authors:** Oliver Blankenstein, Marta Snajderova, Jo Blair, Effie Pournara, Birgitte Tønnes Pedersen, Isabelle Oliver Petit

**Affiliations:** 1 Center for Chronic Sick ChildrenInstitute for Experimental Paediatric Endocrinology, Charité – Universitätsmedizin Berlin, Berlin, Germany; 2 2nd Faculty of MedicineCharles University and University Hospital Motol, Prague, Czech Republic; 3 Alder Hey Children’s NHS Foundation TrustLiverpool, UK; 4 Novo Nordisk Health Care AGZurich, Switzerland; 5 EpidemiologyNovo Nordisk A/S, Søborg, Denmark; 6 Department of Paediatric EndocrinologyHôpital des Enfants, Toulouse, France

## Abstract

**Objective:**

To describe real-life dosing patterns in children with growth hormone deficiency (GHD), born small for gestational age (SGA) or with Turner syndrome (TS) receiving growth hormone (GH) and enrolled in the NordiNet International Outcome Study (IOS; Nbib960128) between 2006 and 2016.

**Design:**

This non-interventional, multicentre study included paediatric patients diagnosed with GHD (isolated (IGHD) or multiple pituitary hormone deficiency (MPHD)), born SGA or with TS and treated according to everyday clinical practice from the Czech Republic (IGHD/MPHD/SGA/TS: *n* = 425/61/316/119), France (*n* = 1404/188/970/206), Germany (*n* = 2603/351/1387/411) and the UK (*n* = 259/60/87/35).

**Methods:**

GH dosing was compared descriptively across countries and indications. Proportions of patients by GH dose group (low/medium/high) or GH dose change (decrease/increase/no change) during years 1 and 2 were also evaluated across countries and indications.

**Results:**

In the Czech Republic, GH dosing was generally within recommended levels. In France, average GH doses were higher for patients with IGHD, MPHD and SGA than in other countries. GH doses in TS tended to be at the lower end of the recommended label range, especially in Germany and the UK; the majority of patients were in the low-dose group. A significant inverse association between baseline height standard deviation score and GH dose was shown (*P* < 0.05); shorter patients received higher doses. Changes in GH dose, particularly increases, were more common in the second (40%) than in the first year (25%).

**Conclusions:**

GH dosing varies considerably across countries and indications. In particular, almost half of girls with TS received GH doses below practice guidelines and label recommendations.

## Introduction

Growth hormone (GH) is approved for the treatment of short stature in children with growth hormone deficiency (GHD) as well as other non-GHD conditions including short children born small for gestational age (SGA) and those with Turner syndrome (TS) (1). Treatment guidelines suggest that for an optimal response, GH should be initiated as early as possible after diagnosis (2, 3, 4). GH dose is individual, based mainly on the recommended dose range and individual response to therapy (2, 3, 4, 5); higher doses may be recommended in some children born SGA who are very short at treatment start (3).

Regular monitoring of GH-treated patients is strongly recommended (2, 3, 4), especially during the first year of treatment when most of the catch-up growth occurs (3) and the growth response is dose-dependent (6, 7). Age and height at treatment start are the strong predictive factors of height gain and adult height (8, 9, 10, 11). Differences in the frequency of monitoring, auxological parameters selected to adjust GH dose and inherent variability among different assays used to assess insulin-like growth factor-I (IGF-I) (12, 13) may result in variable GH dosing patterns among different centres. Further, although GH dosing across countries is guided by the respective disease-specific consensus guidelines (2, 3, 4), differences in national recommendations or insurance reimbursement, as well as other guidance provided by hospital or peer recommendations, or decisions taken on a case-by-case basis may influence GH dosing in clinical practice (14). Dosing may also be affected indirectly by the diagnostic criteria used to evaluate short stature; children who are very short at diagnosis may benefit from a GH dose at the higher end of the dosing range (3).

Two previous surveys evaluated differences in the diagnosis and treatment of patients with GHD across countries and national centres (15, 16). However, both the surveys were published over a decade ago and it is unknown if these differences still exist or if they manifest in other indications such as SGA and TS.

The aim of this report is to describe dosing patterns in real-life clinical practice among GH-treated children with GHD, born SGA or with TS enrolled in the NordiNet International Outcome Study (IOS; ClinicalTrials.gov identifier: Nbib960128).

## Subjects and methods

### Study design

The NordiNet International Outcome study (IOS) is a non-interventional, multicentre study evaluating the long-term effectiveness and safety of Norditropin (somatrophin) (Novo Nordisk A/S) as prescribed by the treating physicians in the real-life clinical setting. The methodology, objectives and study design of NordiNet IOS have been detailed previously (17). Data for the present report were collected prospectively from April 2006 to July 2016; however, study data continued to be collected until December 2016 when the NordiNet IOS concluded. In Germany, prior to 2006, data were collected by the national Novo Nordisk non-interventional study on Norditropin-treated children, GrowthWin. These data were migrated into NordiNet IOS as described in Höybye *et al*. 2013 (17).

### Patient population

Paediatric patients diagnosed with GHD (isolated (IGHD) or multiple pituitary hormone deficiency (MPHD)), born SGA or with TS and treated according to everyday clinical practice from the Czech Republic (IGHD/MPHD/SGA/TS: *n* = 425/61/316/119), France (*n* = 1404/188/970/206), Germany (*n* = 2603/351/1387/411) and the UK (*n* = 259/60/87/35), enrolled in NordiNet IOS and treated with GH for up to 7 years were included in the present analysis. The Czech Republic, France, Germany and the UK are the largest national cohorts in NordiNet IOS. Clinical diagnosis was based on the judgement of the treating physician. Patients were excluded from the present analysis if they did not have valid baseline GH dose information. Patients were categorised as pre-pubertal or pubertal based on the definition for onset of puberty as Tanner breast stage ≥2 in girls and testicular volume ≥4 mL in boys. If puberty stage information was missing, the estimated age of puberty onset was used to impute pubertal status.

### GH dose

GH doses (μg/kg/day) were recorded throughout the patient’s follow-up period (including periods without GH therapy) within the study. The average GH dose for the patient during the full treatment period and duration of GH treatment (years), defined as follow-up period from GH initiation to last reported visit, were calculated. Approved doses recommended in the European Norditropin label are 25–35 µg/kg/day for patients with GHD, 35 µg/kg/day for patients born SGA and 45–67 µg/kg/day for patients with TS (5); based on these data, GH doses prescribed during the study were categorised as low-, medium- and high-dose respectively, for each condition as follows: IGHD and MPHD, ≤25, >25 to ≤35 and >35; SGA, ≤30, >30 to ≤40 and >40; and TS, ≤45, >45 to ≤55 and >55 µg/kg/day. Additionally, change in GH dose from baseline within the first and second year of treatment (two visits, a minimum of 6 months apart) was categorised as a decrease or an increase of 10%, or no change in GH dose, and the proportions of patients by GH dose change category were calculated.

### Statistical analysis

Data are presented as mean, standard deviation (s.d.) and percentages. Descriptive statistics were applied on baseline characteristics (GH dose, duration of GH treatment, age at treatment start, height standard deviation score (HSDS) for national references, body mass index (BMI) standard deviation score (SDS) and IGF-I SDS) by diagnosis and country. Linear regression was performed to analyse the relationship between HSDS at treatment start and GH dose during the full treatment period. Statistical significance was set at *P* < 0.05. Statistical analysis was performed using SAS v9.4 (SAS Institute Inc., Cary, NC, USA).

### Ethics

Informed consent was provided by the parents or guardians of the paediatric patients prior to study enrolment. The study was conducted in accordance with the Declaration of Helsinki and was approved by the local Institutional Ethics Committee/Institutional Review Board and the local regulatory authorities at each study centre and data privacy agencies as required. NordiNet IOS is conducted in accordance with the Good Pharmacoepidemiology Practice guidelines (18).

## Results

### Baseline characteristics

Baseline characteristics of the study population are shown in Table 1. Across all indications, patients in France and the UK had a higher HSDS at baseline than those in the Czech Republic and Germany. Within all indications and across all countries, mean BMI SDS was highest in patients with MPHD. Among patients with GHD (IGHD and MPHD), mean BMI SDS was higher in patients in the UK than in those from the other countries.

**Table 1:** Clinical characteristics of patients with IGHD, MPHD, SGA and TS by country.

**Indication for GH therapy/country**	**Age at treatment start** (years)		**HSDS at GH treatment start**		**BMI SDS at GH treatment start**		**IGF-I SDS at GH treatment start**		Baseline GH dose (µg/kg/day)		**Duration of GH treatment** (follow-up time in study) (years)		**Average GH dose during full treatment period** (µg/kg/day)	
	*n*	Mean (s.d.)	*n*	Mean (s.d.)	*n*	Mean (s.d.)	*n*	Mean (s.d.)	*n*	Mean (s.d.)	*n*	Mean (s.d.)	*n*	Mean (s.d.)
IGHD														
Czech Republic	425	7.9 (4.0)	412	−2.9 (0.9)	413	−0.2 (1.3)	188	−1.7 (1.5)	425	30.1 (6.7)	425	3.2 (2.6)	425	31.7 (6.3)
France	1404	9.2 (4.2)	1375	−2.3 (0.9)	1381	−0.4 (1.2)	703	−1.3 (1.4)	1404	35.8 (7.8)	1404	3.1 (2.8)	1404	36.7 (6.7)
Germany	2603	8.9 (3.8)	2259	−2.7 (0.9)	2254	−0.2 (1.2)	1329	−1.8 (1.5)	2603	28.9 (8.7)	2603	4.3 (2.8)	2603	30.7 (6.4)
UK	259	8.9 (4.6)	244	−2.3 (1.5)	243	0.3 (1.6)	73	−1.6 (1.9)	259	29.7 (9.1)	259	3.4 (2.6)	259	29.7 (6.0)
MPHD														
Czech Republic	61	10.2 (3.4)	59	−2.4 (1.2)	58	0.3 (1.7)	35	−2.9 (1.6)	61	27.5 (7.4)	61	3.8 (2.9)	61	28.8 (6.8)
France	188	9.7 (4.0)	183	−1.9 (1.2)	183	0.1 (1.5)	96	−1.6 (1.7)	188	34.4 (9.8)	188	3.6 (2.6)	188	35.4 (9.6)
Germany	351	9.1 (4.6)	305	−2.5 (1.2)	307	0.2 (1.5)	170	−2.5 (1.9)	351	28.0 (8.9)	351	4.4 (3.3)	351	28.4 (7.3)
UK	60	9.7 (4.3)	55	−1.8 (1.2)	55	1.2 (1.7)	17	−2.5 (3.1)	60	26.7 (5.8)	60	3.7 (2.4)	60	26.1 (5.6)
SGA														
Czech Republic	316	7.2 (3.2)	304	−3.2 (0.7)	304	−0.9 (1.2)	143	−0.6 (1.2)	316	36.5 (9.7)	316	3.6 (2.5)	316	37.1 (6.5)
France	970	8.3 (3.6)	944	−2.8 (0.8)	942	−0.9 (1.2)	407	−0.5 (1.6)	970	43.1 (11.0)	970	3.3 (2.8)	970	43.7 (9.4)
Germany	1387	7.7 (3.0)	1321	−3.1 (0.8)	1328	−0.9 (1.2)	773	−0.9 (1.3)	1387	33.6 (7.0)	1387	4.5 (2.7)	1387	34.7 (5.1)
UK	87	7.1 (3.2)	85	−3.2 (0.9)	85	−0.9 (1.1)	18	−0.6 (1.5)	87	36.9 (10.6)	87	3.3 (2.3)	87	38.1(7.9)
TS														
Czech Republic	119	8.8 (3.7)	115	−2.7 (0.8)	115	0.4 (1.1)	55	−0.6 (1.5)	119	44.9 (6.1)	119	4.4 (3.0)	119	45.3 (5.7)
France	206	8.4 (3.7)	203	−2.3 (1.0)	202	0.4 (1.0)	106	−0.5 (1.3)	206	47.2 (12.3)	206	3.8 (3.0)	206	46.0 (9.7)
Germany	411	8.6 (3.7)	365	−2.9 (0.9)	366	0.4 (1.1)	236	−1.1 (1.4)	411	41.8 (12.5)	411	4.6 (2.8)	411	42.5 (8.3)
UK	35	6.2 (3.9)	33	−2.4 (1.0)	33	0.5 (1.0)	4	−1.8 (1.8)	35	47.7 (12.4)	35	4.2 (3.0)	35	45.1 (10.2)

BMI, body mass index; GH, growth hormone; HSDS, height standard deviation score; IGF-I, insulin-like growth factor-I; IGHD, isolated growth hormone deficiency; MPHD, multiple pituitary hormone deficiency; N, number of patients; SGA, small for gestational age; s.d., standard deviation; SDS, standard deviation score; TS, Turner syndrome.

Differences in the mean age at treatment start were observed across countries and across all indications. Among patients with IGHD, those in the Czech Republic, where mean age at treatment start was 7.9 years, were on average more than 1 year younger at treatment start than those in the other three countries. Conversely, patients with MPHD in the Czech Republic, where the mean age at treatment start was 10.2 years, were slightly older at treatment start than those in France, Germany or the UK, where mean age at treatment start was between 9.1 and 9.7 years of age. Patients born SGA in France were on average 8.3 years of age at treatment start, which was older than those in the Czech Republic, Germany and the UK. Girls with TS in the UK, where the mean age at treatment start was 6.2 years, were on average more than 2 years younger at treatment start than those in the other countries.

Across all indications, duration of treatment (follow-up period in the study) tended to be longer in patients in Germany compared with the other countries, despite age at treatment start being similar between Germany and the other countries; this is probably a reflection of the earlier date of inclusion for German patients whose data were migrated into NordiNet IOS from GrowthWin.

At baseline, patients in France diagnosed with IGHD, MPHD or born SGA received higher GH doses than in the other countries. In patients with TS, GH doses were lower in Germany than in the other countries.

### Average GH dose during each treatment year

**Figure 1:**
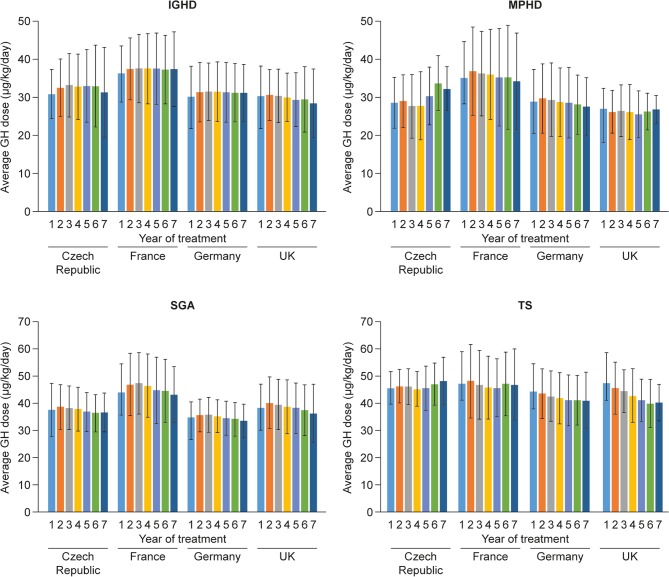
Average GH dose by treatment year during the GH treatment period, by indication and country. Data are mean (s.d.). GH, growth hormone; IGHD, isolated growth hormone deficiency; MPHD, multiple pituitary hormone deficiency; s.d., standard deviation; SGA, small for gestational age; TS, Turner syndrome.

For patients with IGHD, MPHD or SGA, the average GH dose in each treatment year was higher among children in France than in the other three countries (Fig. 1). For patients with IGHD and MPHD, the lowest mean average doses were observed in the UK, and for those born SGA, the lowest mean average GH doses were observed in Germany. Mean average GH doses among patients with TS were similar between the Czech Republic and France, showing a trend to be lower with increasing duration of treatment in both the UK and Germany. Importantly, in Germany, the mean average GH doses were lower than that recommended in the label for girls with TS (<45 µg/kg/day) throughout the study.

### Proportion of patients in low-, medium- and high-GH-dose groups (based on average GH dose during the full treatment period) by indication and country

**Figure 2:**
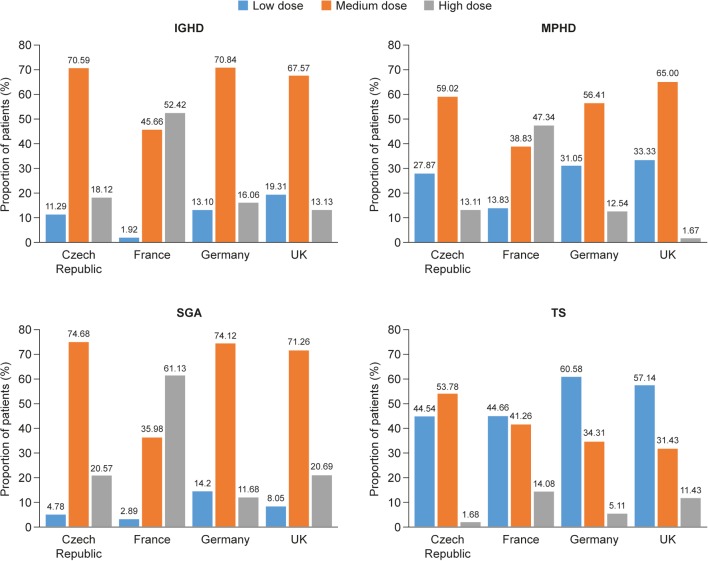
Proportion of patients in low-, medium- and high-GH-dose groups (based on average GH dose during the full treatment period) by indication and country. Low-, medium- and high-dose groups are as follows: IGHD and MPHD, ≤25, >25 to ≤35 and >35 respectively; SGA, ≤30, >30 to ≤40 and >40 respectively; TS, ≤45, >45 to ≤55 and >55. GH, growth hormone; IGHD, isolated growth hormone deficiency; IOS, International Outcome Study; MPHD, multiple pituitary hormone deficiency; s.d., standard deviation; SGA, small for gestational age; TS, Turner syndrome.

Across all indications, Germany and the UK had the highest proportions of patients in the low-dose group, with many patients being dosed below label recommendations (Fig. 2). Proportionally more patients in France with IGHD, MPHD or SGA received GH doses within the high-dose range than in the other countries; the majority of these patients in the Czech Republic, Germany and the UK received GH doses within the medium GH dose range. In the Czech Republic and France, similar proportions of girls with TS received GH doses in the low- and medium-dose ranges. In contrast, in Germany and the UK, the majority of girls with TS received GH doses below both practice guidelines and European label recommendations.

### Relationship between HSDS and average GH dose during treatment

Across all indications and for most countries, a statistically significant association (*P* < 0.05) was observed between baseline HSDS and average GH dose given to the patient during the full treatment period, with the shortest patients receiving the highest doses (Table 2). No significant association between HSDS and average GH dose was found for patients with IGHD or TS in the Czech Republic, for patients born SGA in France, or for patients born SGA or with TS in the UK; however this may be due to the small number of observations.

**Table 2:** Results from linear regression analysis of the association between baseline HSDS and GH dose during the full treatment period.

	**Association between baseline HSDS and GH dose**		
**Indication for GH therapy/country**	Estimated effect	s.e.m.	P*
IGHD			
Czech Republic	−0.0111	0.0070	0.1144
France	−0.0098	0.0038	0.0093
Germany	−0.0156	0.0032	<0.0001
UK	−0.0664	0.0152	<0.0001
MPHD			
Czech Republic	−0.0953	0.0191	<0.0001
France	−0.0414	0.0086	<0.0001
Germany	−0.0472	0.0096	<0.0001
UK	−0.0611	0.0297	0.0443
SGA			
Czech Republic	−0.0159	0.0069	0.0214
France	−0.0048	0.0029	0.0971
Germany	−0.0193	0.0041	<0.0001
UK	−0.0211	0.0119	0.0803
TS			
Czech Republic	−0.0047	0.0133	0.7252
France	−0.0161	0.0073	0.0283
Germany	−0.0140	0.0057	0.0135
UK	−0.0043	0.0168	0.8018

* Statistical significance of association.

GH, growth hormone; IGHD, isolated growth hormone deficiency; MPHD, multiple pituitary hormone deficiency; SGA, small for gestational age; HSDS, height standard deviation score; s.e.m., standard error of the mean; TS, Turner syndrome.

### GH dose changes

**Figure 3:**
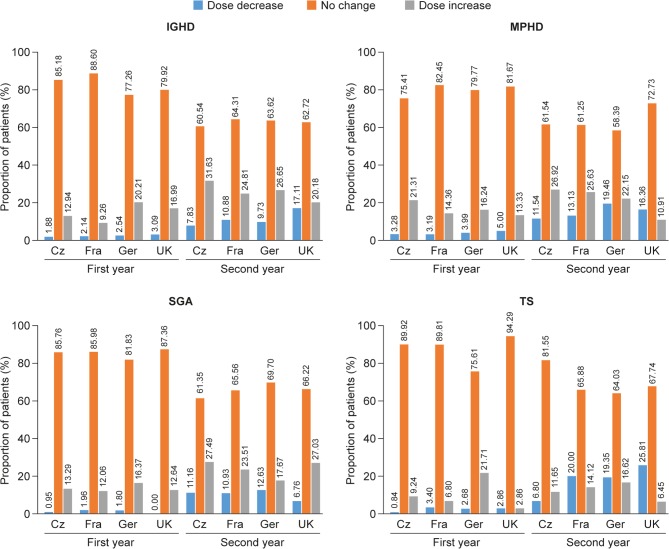
Proportion of patients with decreasing, no change, or increase in GH dose during the first and second year of treatment by indication and country. Increase or decrease of GH dose by >10%. Cz, Czech Republic; Fra, France; Ger, Germany; GH, growth hormone; IGHD, isolated growth hormone deficiency; MPHD, multiple pituitary hormone deficiency; s.d., standard deviation; SGA, small for gestational age; TS, Turner syndrome.

During the first year, GH dose was unchanged for more than 75% of patients across all countries for those with IGHD, MPHD or SGA; this proportion fell to approximately 60% of patients during year 2 (Fig. 3). On the whole, apart from patients with TS during year 2, more increases than decreases in GH dose were reported for proportionally more patients during both treatment years. With the exception of patients with TS in Germany and those with MPHD in the UK, the proportions of patients with an increase in GH dose in year 2 exceeded those with an increase in dose in year 1. Considering year 2 of treatment, with the exception of patients born SGA, a lower proportion of patients in the UK had an increase in GH dose than in the Czech Republic, France or Germany.

When patients were categorised by pubertal status (Supplementary Fig. 1, see section on Supplementary data given at the end of this article) variable patterns of GH dose titration from pre-puberty to puberty were observed. For patients with IGHD in the Czech Republic and France, some up-titration of GH dose was observed as reflected in an increase in the proportion of patients in the high-dose group and a decrease in the proportion of patients in the low-dose group. This trend was also observed for patients born SGA in France. The opposite trend was observed for patients with MPHD or born SGA treated in the UK, with an increase in the proportion of patients in the low GH dose group when comparing pre-pubertal and pubertal patients.

## Discussion

This study provides a detailed description of GH dosing patterns in everyday clinical practice in children enrolled in NordiNet IOS in the Czech Republic, France, Germany and the UK, diagnosed with IGHD, MPHD, born SGA or with TS. As this is a cross-sectional study, it is not possible to investigate any association between trends in GH dosing patterns and clinical outcomes. Therefore, the focus of this report is to describe treatment patterns across indications and by country to determine if these are aligned with, or diverge from international treatment guidelines and the Norditropin product label for Europe.

To our knowledge, these results are the first to document that, during the period of the study, differences exist in real-life clinical practice in GH dosing patterns among the four countries evaluated. Overall, we showed that in the Czech Republic, GH dosing was generally within recommended levels across all indications. However, although mean age at treatment start for patients with IGHD in the Czech Republic was 7.9 years, which was at least 1 year lower than in the other three countries, the average age at GH treatment start for patients with MPHD or TS in the Czech Republic was higher (mean age of 10.2 and 8.8 years respectively) than in the three comparator countries. In France, GH doses were generally higher than in the other countries for patients with IGHD, MPHD or born SGA. Furthermore, mean age at treatment start for patients with IGHD (9.2 years) or SGA (8.3 years) in France was higher than in the remaining three countries. In patients with TS, GH doses tended to be at the lower end of the recommended range, especially in Germany and the UK; of interest is that patients with TS in the UK were on average two years younger at treatment start than patients in the Czech Republic, France and Germany (6.2 vs 8.4–8.8 years). On the whole, with some exceptions across indications and countries, we found an inverse association between HSDS at baseline and GH dose, with the shortest children generally receiving the highest GH doses.

In patients with IGHD and MPHD, we observed variations among countries in baseline and average GH dose, as well as in age and height at start of treatment. In France, patients had the highest mean HSDS at baseline and as discussed above were, on average, older than those in the other countries, possibly reflecting national differences in the diagnostic criteria for growth retardation, as well as the criteria for initiating GH treatment. Indeed, a recent publication has highlighted the impact of outdated national growth references (19), as found in France, on the diagnosis of short stature, indicating that the proportions of children with short stature are likely to be underestimated, and that children are likely to be diagnosed later, in countries using outdated national growth references compared with countries using more recent national growth references or the World Health Organization growth references/standards (20, 21, 22, 23, 24). In other countries, such as the UK, GH may be initiated in children with biochemical evidence of GHD and evidence of growth retardation, despite HSDS ≥−2; this is important as it permits early diagnosis and GH treatment initiation for children with conditions such as septo-optic dysplasia or midline tumours/cranial irradiation.

In France, average GH doses were at the higher end of the label range, which may reflect historical differences between France and the other countries in the approved GH dose; prior to harmonisation of this dose, 50 µg/kg/day was the nationally recommended dose for GH therapy in short children born SGA in France, with a lower dose (35 μg/kg/day) approved in the rest of Europe. The higher GH doses reported in France in this study may therefore reflect that some physicians in France continue to prescribe higher GH doses. For patients with TS, baseline and average GH doses were generally at the lower end of the recommended GH dose range across countries, with almost half of these patients receiving doses below the label recommendations. Treatment with lower-than-recommended GH doses in girls with TS and children with SGA have previously been reported in the UK cohort of Kabi International Growth Study (KIGS; 25), the Italian cohort of Genetics and Neuroendocrinology of Short Stature International Study (GeNeSIS; 26) and the first report from the PAtients TReated with Omnitrope® ( PATRO) registry (27).

While it is established that GH dosing in girls with TS should be optimised to promote growth and normalise adult height, it is equally recognised that the dose should be adapted according to the patient’s growth response (28) and IGF-I levels (9, 29). In addition, as it is recognised that girls with TS are at an increased risk of dyslipidaemia, heart problems (including arrhythmia, hypertension atherosclerosis and aortic dilatation) and have impaired nonverbal skills compared with normal girls (29), the impact of GH therapy on glucose metabolism, cardiovascular abnormalities and cognitive function warrants further investigation. Although GH treatment may have an adverse effect on insulin sensitivity (30), data support that GH treatment may reduce abdominal adiposity and improve glucose tolerance in girls with TS suggesting that the beneficial effects of GH on body composition and regional fat deposition may outweigh the transient insulin antagonism associated with GH administration (31). As discussed above, optimisation of clinical outcomes in children treated with GH requires frequent monitoring of clinical endpoints – effectiveness (height velocity or HSDS; IGF-I SDS) and safety (occurrence of adverse events) – with subsequent adjustment of GH dose as required. Adequate dose titration appears to be especially important in the first years of treatment (6, 7), but it is less clear if higher GH doses during puberty translate to improved height outcomes, with some studies showing significant benefits (i.e. in patients with GHD) (32) and others showing no difference (i.e. in those born SGA) (33). In the present study, we observed variable patterns of dose titration among countries and across indications during the first 2 years of treatment and during puberty. For patients with IGHD, MPHD and SGA dose changes were more common during year 2 than year 1. A trend to treat greater proportions of children with a higher dose during transition through puberty was observed in the Czech Republic for IGHD, MPHD and SGA. In contrast, in the UK and Germany, proportionally fewer patients than in the other countries received higher GH doses on entering puberty. Overall, the real-world data collected in NordiNet IOS indicate that there are national specific practices in treating patients with GH, which are only partly explicable (e.g. national guidelines, absence of updated growth charts). It remains to be established whether a proportion of patients receiving lower-than-recommended GH doses may have benefited from an increase in dose or whether a proportion were dosed appropriately, i.e. meeting their individualised growth target. In addition to height gain, which is recognised to be improved with earlier GH treatment start, the benefits of GH treatment on metabolic and other endpoints (34, 35), including cognitive outcomes (36), may be dependent on appropriate dosing and age-appropriate treatment start.

### Strengths and limitations

The large size of the study cohort and the length of the follow-up period have permitted meaningful comparisons of GH dosing and changes in GH dose across the individual countries and indications. However, the heterogeneous patient population, and differences in reporting standards between clinics and across countries, limit the power of the study results to draw firm conclusions regarding potential underlying reasons for variations in GH dosing among the countries studied. Further, due to the observational nature of the study, potential selection biases cannot be ruled out.

## Conclusion

This evaluation of GH dosing patterns among children with IGHD, MPHD, SGA and TS enrolled in NordiNet IOS in the Czech Republic, France, Germany and the UK highlights differences in clinical practice among these countries. The study results draw attention to variations in GH dosing across countries and indications, with some patients receiving GH doses that are below the label recommendations, and raises awareness that current practices may diverge from European or international clinical practice guidelines or the European product label recommendation. Of particular concern is the finding that across all countries, nearly half of girls with TS received GH doses lower than the recommended label. Further studies are warranted to investigate the underlying causes that represent potential barriers to the current guidelines.

## Supplementary Material

Supporting Figure 1

## Declaration of interest

O Blankenstein received honoraria from Novo Nordisk and payment for his role as a member of the International Steering Committee for NordiNet IOS. M Snajderova received honoraria from Novo Nordisk and payment for her role as a member of the International Steering Committee for NordiNet IOS. J Blair received honoraria from Novo Nordisk and payment for her role as a member of the International Steering Committee for NordiNet IOS. E Pournara and B Tønnes Pedersen are employees of Novo Nordisk. I Oliver Petit received honoraria from Novo Nordisk and payment for her role as a member of the International Steering Committee for NordiNet IOS.

## Funding

This project was sponsored by Novo Nordisk Healthcare AG, Zürich, Switzerland.

## Author contribution statement

All authors made substantial contributions to conception and design, and/or acquisition of data, and/or analysis and interpretation of data; participated in drafting the article or revising it critically for important intellectual content; and gave final approval of the version to be submitted.
