# Supplementary material for: Real-life GH dosing patterns in children with GHD, TS or born SGA: a report from the NordiNet® International Outcome Study
Source: Eur J Endocrinol. 2017 May 18;177(2):145–55. doi: 10.1530/EJE-16-1055 (PMC5488395; doi:10.1530/EJE-16-1055)
Supplement: Supporting Figure 1 [file eje-177-145-s001.pdf]

Manuscript ID no.: EJE-16-1055.R1

Title: Real-life GH dosing patterns in children with GHD, TS or born SGA: a report from the NordiNet® International Outcome Study

Authors: Oliver Blankenstein[1], Marta Snajderova[2], Jo Blair,[3] Effie Pournara[4], Birgitte Tønnes Pedersen[5] and Isabelle Oliver Petit[6]

SUPPLEMENTARY MATERIAL

Supplementary Figure 1 Proportion of patients in low-, medium- and high- GH dose groups by indication, country and pubertal status

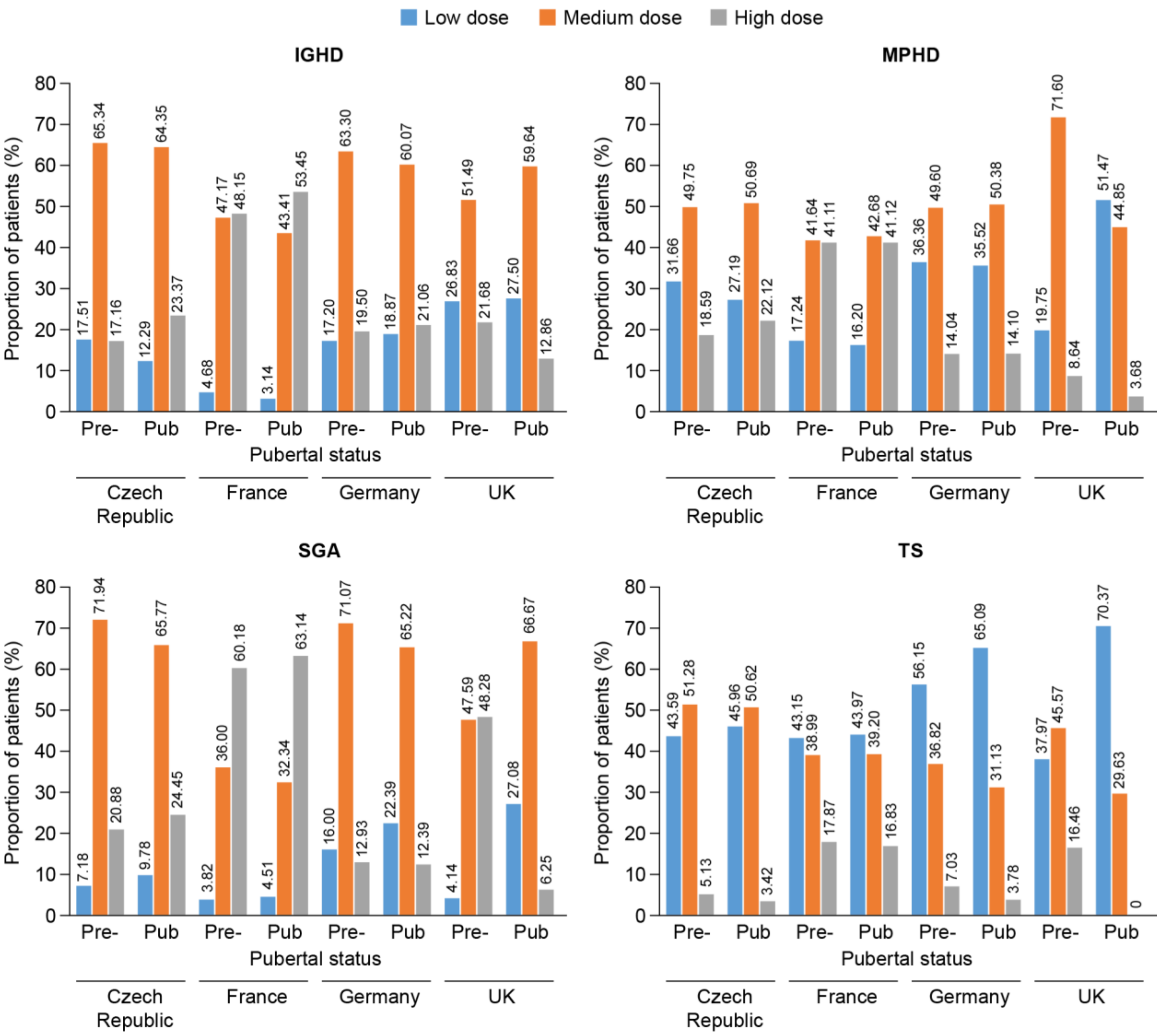

Low-, medium- and high-dose groups are as follows: IGHD and MPHD, ≤25, >25–≤35 and >35, respectively; SGA, ≤30, >30–≤40 and >40, respectively; TS, ≤45, >45–≤55 and >55. GH, growth hormone; IGHD, isolated growth hormone deficiency; MPHD, multiple pituitary hormone deficiency; Pre-, pre-pubertal; Pub, pubertal; SGA, small for gestational age; TS, Turner syndrome.
